# HIBISCUS trial (hernia incidence with continuous barbed vs interrupted suturing in colorectal surgery with small incisions): a contemporary study protocol for a multicenter randomized controlled trial

**DOI:** 10.1186/s12893-026-03528-5

**Published:** 2026-01-26

**Authors:** Yushi Yamakawa, Hiroki Takahashi, Kazuyoshi Shiga, Takahisa Hirokawa, Nozomu Nakai, Koshiro Harata, Hajime Ushigome, Takuya Suzuki, Akira Kato, Shuhei Uehara, Hiroyuki Asai, Junki Kato, Misato Sawai, Takahiro Otani, Tatsuhito Yamamoto, Shuji Takiguchi

**Affiliations:** 1 Department of Gastroenterological Surgery, Nagoya City University Graduate School of Medical Sciences, 1 Kawasumi, Mizuho-cho, Mizuho-ku, Nagoya, Aichi 467- 8601 Japan; 2 Department of Gastroenterological Surgery, Nagoya City University West Medical Center, 1-1-1 Hirate-cho, Kita-ku, Nagoya, Aichi 462-0057 Japan; 3 Department of Gastroenterological Surgery, Nagoya City University East Medical Center, 1-2-23 Wakamizu, Chikusa-ku, Nagoya, Aichi 464-8547 Japan; 4 Department of Gastroenterological Surgery, Kariya Toyota General Hospital, 5-15 Sumiyoshi-cho, Kariya, Aichi 448-8505 Japan; 5 Department of Gastroenterological Surgery, Toyota Memorial Hospital, 1-1 Heiwa-cho, Toyota, Aichi 471-0821 Japan; 6 Department of Gastroenterological Surgery, Toyokawa City Hospital, 23 Noji, Hachiman-cho, Toyokawa, Aichi 442-8561 Japan; 7 Division of Cancer Information and Control, Department of Preventive Medicine, Aichi Cancer Center Research Institute, 1-1 Kanokoden, Chikusa-ku, Nagoya, Aichi 464-8681 Japan; 8 Department of Radiology, Nagoya City University Graduate School of Medical Sciences, 1 Kawasumi, Mizuho-cho, Mizuho-ku, Nagoya, Aichi 467-8601 Japan

**Keywords:** Incisional hernia, Continuous barbed suturing, Interrupted suturing, Randomized controlled trial

## Abstract

**Background:**

Incisional hernia is the most common long-term complication after abdominal surgery, including minimally invasive colorectal procedures. In Japan, fascial closure of small midline incisions is generally performed using interrupted suturing, but high-quality evidence comparing continuous barbed suturing with interrupted suturing is limited. Continuous closure may reduce the risk of incisional hernia; however, robust randomized data in laparoscopic and robot-assisted colorectal surgery are lacking. This study aims to determine whether continuous barbed suturing is superior to conventional interrupted suturing in preventing incisional hernia after minimally invasive colorectal cancer surgery.

**Methods:**

The HIBISCUS trial is a multicenter, prospective, open-label, randomized controlled study comparing continuous barbed suturing with interrupted monofilament suturing for fascial closure of mini-laparotomy incisions. Eligible adults with colorectal cancer undergoing elective laparoscopic or robot-assisted resection will be randomized 1:1 to continuous closure using a barbed absorbable suture (STRATAFIX Symmetric PDS Plus; bite width of 5 mm and stitch spacing of 5 mm) or interrupted closure using a monofilament absorbable suture (PDS Plus; bite width of 8 mm and stitch spacing of 8 mm). Only board-certified surgeons who complete a standardized pre-trial suturing competency assessment will be allowed to participate. The primary endpoint is the incidence of incisional hernia within 18 months after surgery, assessed mainly by scheduled abdominal CT imaging, with clinical examination as complementary. Secondary endpoints include fascial closure time, postoperative wound complications (including surgical site infection (SSI)), postoperative wound pain, and reoperation or readmission. All CT images will be independently reviewed by two blinded specialists. Based on power calculations, 422 patients will be enrolled.

**Discussion:**

This trial will be the first randomized study to directly compare continuous barbed suturing with conventional interrupted suturing for small midline incisions in minimally invasive colorectal surgery. By standardizing suturing parameters and requiring surgeon competency verification, the HIBISCUS trial is designed to yield high-quality evidence on optimal fascial closure technique. The results are expected to influence clinical practice, inform guideline development, and contribute to the global standardization of abdominal wall closure practices.

**Trial registration:**

This study is registered in the Japan Registry of Clinical Trials (jRCT1040250129). Registration date: November 21, 2025.

**Supplementary Information:**

The online version contains supplementary material available at 10.1186/s12893-026-03528-5.

## Background

Midline laparotomy has remained a workhorse incision from the open era through the widespread adoption of laparoscopy and robotics because it enables rapid entry to the peritoneal cavity while minimizing injury to abdominal wall nerves, vessels, and muscle. Nevertheless, incisional hernia (IH) is the most common complication of midline incisions, with reported incidences of approximately 10–20% [1–5]. Rates are even higher—around 30–35%—in high-risk groups such as patients with obesity or abdominal aortic aneurysm [6–8]. IH causes pain, functional limitations, and reduced quality of life and may lead to serious complications including bowel obstruction and strangulation [5]. The public health burden is substantial: in the United States an estimated 348,000 IH repairs are performed annually, with related costs approaching 3.2 billion USD [9, 10]. Preventing IH is therefore a priority in abdominal surgery.

Currently, the most widely practiced suturing methods for midline laparotomy closure are interrupted suturing (IS) and continuous suturing. IS provides multiple anchoring points by closing the abdominal wall stitch by stitch, offering greater stability and reducing the risk of loss of fascial integrity. However, compared with continuous suturing, it requires more time and makes it difficult to achieve uniform tension at each knot. In contrast, continuous suturing allows faster closure with more even distribution of tension but carries disadvantages such as the risk of tissue ischemia due to excessive tightening and the possibility of wound failure if the suture unravels at a single point. Barbed sutures have been developed as novel materials designed to overcome these disadvantages.

Over the past several decades, suture strategies for closing midline laparotomy have been rigorously evaluated. Meta-analytic data suggest that continuous suturing with monofilament (non-barbed) sutures reduces IH compared with IS [5, 11]. In emergency settings such as fecal peritonitis and other contaminated abdominal procedures, a prospective multicenter randomized trial demonstrated that fascial closure with triclosan-coated barbed sutures reduced the rate of wound dehiscence (evisceration) compared with standard loop closure techniques [12].

Since Jacobs et al. first reported laparoscopic colectomy in 1991, minimally invasive surgery with a small abdominal incision has gradually become the standard approach for colorectal cancer in the 2000s [13]. Weber et al. first reported telerobotic-assisted laparoscopic right and sigmoid colectomies for benign disease in the early 2000s [14]. Soon thereafter, Hashizume and Tsugawa described three colonic resections for malignant disease performed with a robotic system [15, 16]. In Japan, robotic surgery has been reimbursed under the national health insurance system since 2018 for rectal cancer and since 2022 for colon cancer, and its utilization has shown a steady annual increase. In laparoscopic and robotic surgery, the midline incision is smaller than in open surgery, and several reports have suggested that the incidence of IH is therefore lower compared with open procedures [17, 18]. As minimally invasive approaches have reduced incision size, IH rates after laparoscopic surgery appear lower than in the open era; even so, IH after laparoscopic midline extraction closed with IS has been reported in the range of 5–13% [17, 19–21]. Pyo et al. reported an IH rate of 1.3% (1 out of 80 patients) at 12 months following abdominal fascial closure using a monofilament polydioxanone barbed suture (MONOFIX^®^) in their prospective single-arm clinical trial [22]. In addition, recent experimental and clinical studies have provided further insight into the contemporary use of barbed sutures. Experimental data suggest that the material properties of barbed sutures may contribute to stable tissue approximation and maintenance of tensile integrity during the healing process, and recent multicenter clinical data indicate that barbed sutures do not increase the risk of abdominal wall dehiscence compared with conventional sutures after laparotomy [23, 24]. These recent findings reflect a growing shift in fascial closure strategies in the era of minimally invasive and robotic colorectal surgery, highlighting the increasing clinical relevance of barbed sutures in contemporary practice.

IH following midline laparotomy has long been a focus of surgical research. While considerable knowledge exists regarding patient-related risk factors and the role of suture materials, technical aspects—particularly the influence of suture technique—have been less extensively evaluated [5, 11, 25]. Current evidence from randomized trials and meta-analyses indicates that mass closure using continuous suturing provides the most effective method for preventing IH in midline incisions. Compared with layered closure with IS, this approach is both technically simpler and faster to perform [25, 26]. In addition, the use of slow absorbable sutures rather than non-absorbable materials has been associated with lower rates of IH, reduced postoperative pain, and fewer wound infections [11, 25, 27].

The optimal, generalizable closure technique for contemporary small midline incisions, particularly in the setting of laparoscopic and robotic colorectal surgery—remains unsettled. To our knowledge, there has been no multicenter randomized controlled trial (RCT) directly comparing continuous suturing and IS techniques for midline mini-laparotomy closure in the setting of laparoscopic or robotic colorectal surgery. Given the persistent burden of IH and the suggestive but heterogeneous evidence favoring continuous barbed suturing (CBS), further protocolized evaluation is warranted.

Given the lack of high-quality evidence, we designed a multicenter randomized controlled trial to address this issue. This study aims to determine whether CBS is superior to IS in the setting of laparoscopic and robotic surgery. To our knowledge, this will be the first randomized controlled trial to evaluate the use of CBS in this context, and its findings are expected to make a significant contribution to the advancement of global surgical practice.

## Current status of midline mini-laparotomy closure in Japan (based on our original survey)

In Japan, fascial closure of midline incisions has traditionally been performed using IS, which has long been regarded as the standard method for midline fascial closure. In our nationwide survey conducted in 2024 across 50 institutions, 3 institutions (6%) reported using continuous suturing, 45 institutions (90%) used IS, and 2 institutions (4%) reported using both techniques. The survey also revealed that, in cases where IS was applied, more than half of the institutions (58%) responded that the knot tying was performed by surgeons with less than three years of surgical experience. This suggests that the involvement of relatively inexperienced surgeons in knot tying may contribute to the occurrence of IH through loosening of knots in IS.

Although several retrospective studies from Japan have examined the incidence of IH after laparoscopic colorectal surgery, none have directly compared continuous and IS for fascial closure. Consequently, the evidence base remains limited [19, 21]. As a result, continuous suturing has not yet been widely adopted in Japan in the context of laparoscopic or robotic colorectal surgery.

At Nagoya City University, midline incisions in colorectal surgery are closed using continuous suturing with STRATAFIX Symmetric PDS Plus, a barbed suture. STRATAFIX Symmetric PDS Plus, manufactured by Ethicon (Johnson & Johnson, Raritan, NJ, USA), is a barbed suture that does not require knot tying. By incorporating anchoring points to prevent loosening and eliminating the need for continuous traction, it addresses the main drawback of conventional continuous suturing—loss of tension along the suture line—which can lead to the development of IH. In our institution, among 215 cases performed between June 2022 and December 2023, the incidence of IH at one year postoperatively was 3.5%.

## Objective

The objective of the HIBISCUS trial is to compare CBS with IS performed using a monofilament suture. The overall aim of this study is to reduce the incidence of IH, which is one of the most common complications after abdominal surgery. We hypothesize that CBS will significantly decrease the rate of IH, thereby reducing patient morbidity, improving quality of life, and substantially lowering healthcare costs.

The primary endpoint is the incidence of IH within 18 months after surgery, primarily assessed using scheduled abdominal CT imaging performed at predefined follow-up time points in all patients, with clinical examination serving as a complementary assessment. Secondary endpoints include fascial closure time, postoperative complications (particularly SSI), postoperative wound pain, and the rate of reoperation or readmission.

## Methods/design

### Trial design

The HIBISCUS trial is designed as a prospective, multicenter, open-label RCT comparing CBS with IS.

**Fig. 1:**
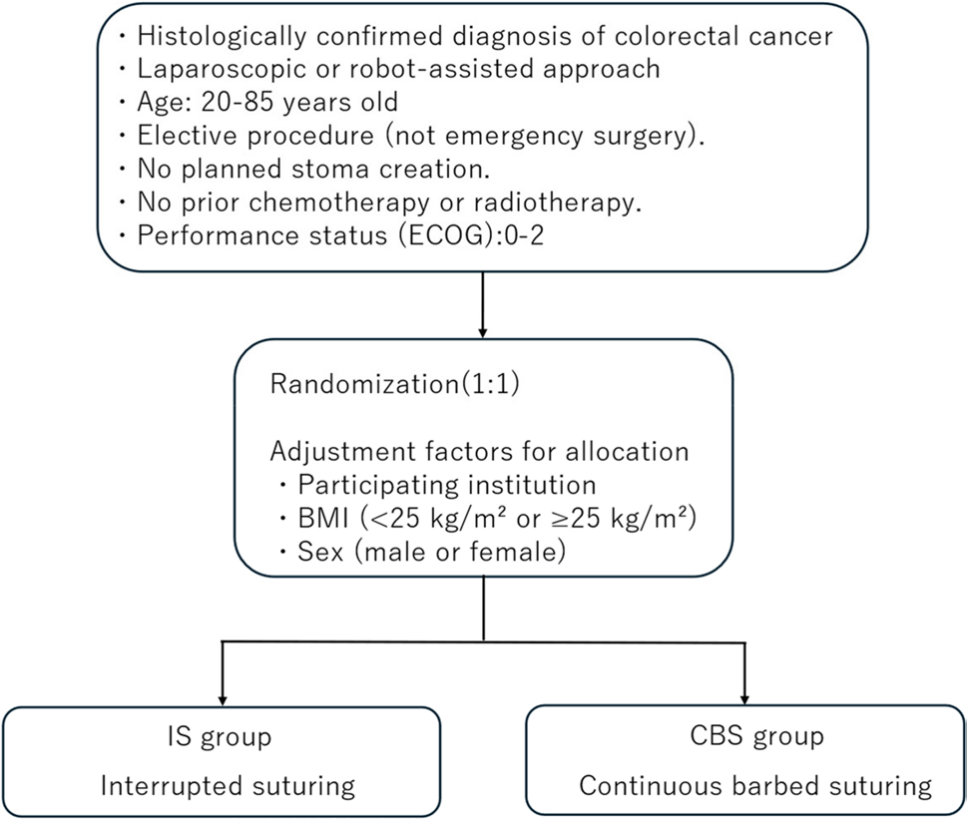
Study design of the HIBISCUS trial. ECOG Eastern Cooperative Oncology Group, IS Interrupted Suturing, CBS Continuous Barbed Suturing

The HIBISCUS trial is a multicenter, prospective, open-label, randomized controlled study comparing continuous barbed suturing with interrupted monofilament suturing for fascial closure of mini-laparotomy incisions (Fig. 1). The study is conducted across six participating institutions in Aichi Prefecture, Japan. Eligible adults with colorectal cancer undergoing elective laparoscopic or robot-assisted resection will be randomized in a 1:1 ratio to continuous closure using a barbed absorbable suture (STRATAFIX Symmetric PDS Plus; bite width of 5 mm and stitch spacing of 5 mm) or interrupted closure using a monofilament absorbable suture (PDS Plus; bite width of 8 mm and stitch spacing of 8 mm). This study is registered in the Japan Registry of Clinical Trials (jRCT1040250129). Registration date: November 21, 2025. Ethical approval was obtained from the Certified Review Board of Nagoya City University, and the protocol has been reviewed and approved by the institutional review boards of all participating hospitals. Patients will be recruited from six hospitals in Aichi Prefecture. The planned enrollment period is 1.5 years, followed by a 1.5-year follow-up period, resulting in a total maximum follow-up duration of 3 years. The study protocol was developed in accordance with the Standard Protocol Items: Recommendations for Interventional Trials (SPIRIT) 2013 guidelines.

### Patient and public involvement

Patients and the public were not involved in the design, conduct, reporting, or dissemination plans of this research, consistent with the SPIRIT 2013 recommendations.

### Study population

Patients with a pathological diagnosis of colorectal cancer who are scheduled to undergo robotic or laparoscopic surgery in which a midline incision will be used for specimen extraction will be eligible for inclusion.

### Inclusion criteria

Patients diagnosed with colorectal cancer and scheduled for elective laparoscopic or robotic-assisted surgery requiring a small midline incision will be asked to provide informed consent at the outpatient clinic or hospital prior to surgery. Eligible patients must meet all of the following criteria:

- Scheduled to undergo elective laparoscopic or robotic-assisted colorectal surgery requiring a mini-laparotomy with a small midline incision for specimen extraction.
- Histologically confirmed diagnosis of colorectal cancer (adenocarcinoma, mucinous carcinoma, signet-ring cell carcinoma, or adenosquamous carcinoma) based on preoperative biopsy.
- Eastern Cooperative Oncology Group (ECOG) performance status of 0–2.
- Age 20–85 years at the time of enrollment.
- No planned stoma creation.
- No history of neoadjuvant chemotherapy or radiotherapy.
- Provision of adequate study explanation and written informed consent.

### Exclusion criteria

- Presence of any of the following severe comorbid conditions:
  - ⚬ Active systemic infection requiring treatment.
  - ⚬ Fever ≥ 38.0℃ at the time of enrollment.
  - ⚬ Ongoing systemic administration (oral or intravenous) of steroids or other immunosuppressive agents.
  - ⚬ Uncontrolled diabetes mellitus.
  - ⚬ Unstable angina (onset within the past 3 weeks or recent worsening of symptoms) or history of myocardial infarction within the past 6 months.
  - ⚬ Uncontrolled valvular disease, dilated cardiomyopathy, or hypertrophic cardiomyopathy.
- Peritoneal dissemination.
- History of midline laparotomy with a current or previous IH.
*Note: A history of midline laparotomy alone is not an exclusion criterion.*
- Psychiatric disorders or symptoms that interfere with daily activities.
- Pregnancy or possibility of pregnancy.
- Any other condition deemed inappropriate for participation in this study by the principal or sub-investigator.

### Registration procedure

The Research Electronic Data Capture (REDCap) platform is used as the electronic data capture system for this trial and is managed by the Data Management Center of Nagoya City University. After written informed consent is obtained, eligible patients are registered in the REDCap system. The registration record includes the study identification number, date of birth, sex, responsible physician, and confirmation of eligibility. Secure access to the system is ensured by assigning each participating institution a unique login ID and password with restricted user permissions.

### Randomization procedure

Randomization is performed within the same REDCap platform using a stratified block randomization method. Stratification factors include participating institution, body mass index (BMI; <25 kg/m² or ≥ 25 kg/m²), and sex (male or female), as BMI and sex are established risk factors for incisional hernia and may influence abdominal wall characteristics and wound healing. A fixed block size is used within each stratum to ensure balance between the treatment groups. Once patient eligibility is confirmed in REDCap, allocation to either the continuous barbed suturing (CBS) group or the interrupted suturing (IS) group is automatically generated by the system. This automated process ensures allocation concealment and minimizes the potential for investigator-related bias in treatment assignment.

### Interventions

**Fig. 2:**
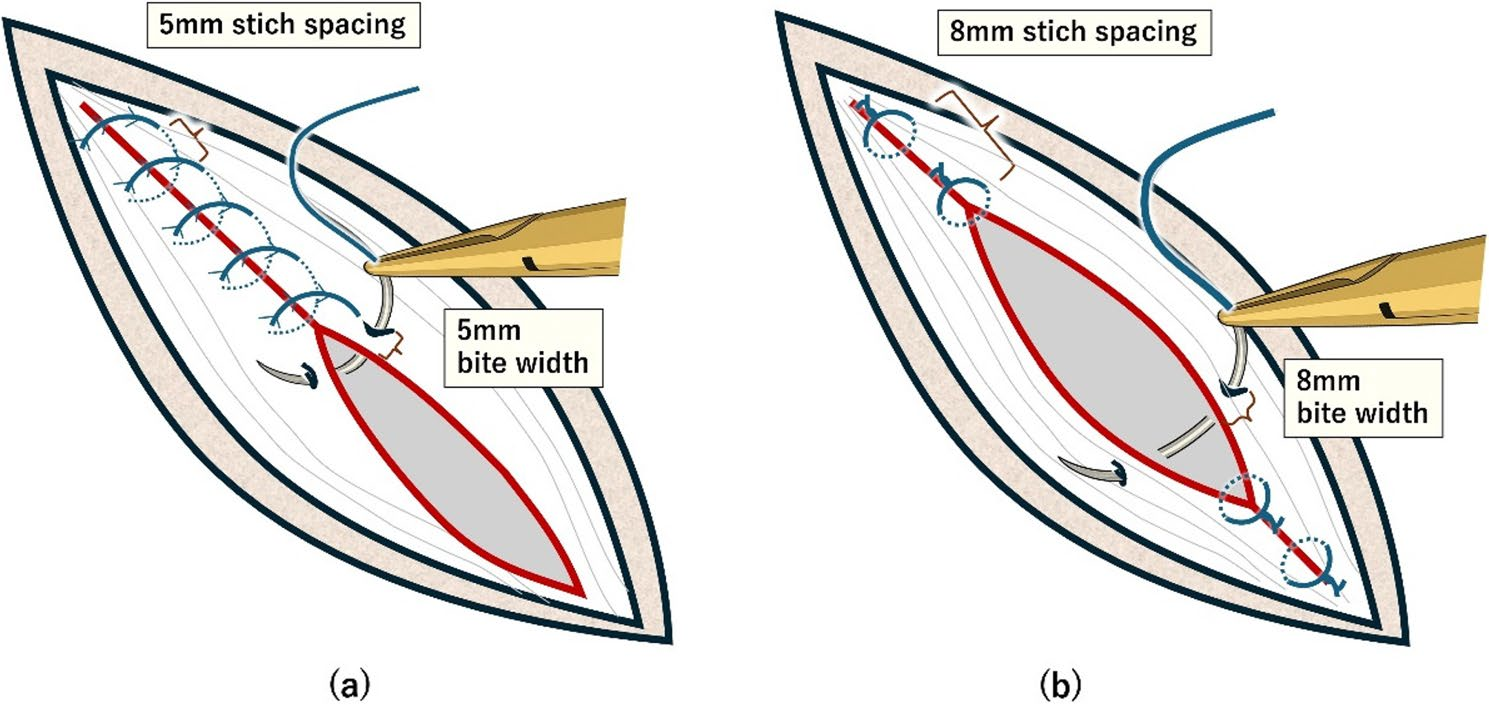
Schematic Illustration of Fascial Closure (**a**) continuous barbed suturing, (**b**) interrupted suturing

In this trial, IS will be compared with CBS. In the IS group, PDS Plus, 45 cm, CTB-1 needle, size 0 will be used with bite widths of 8 mm and suture spacing of 8 mm. In the CBS group, STRATAFIX Symmetric PDS Plus, 45 cm, CTB-1 needle, size 1 will be used with bite widths of 5 mm and suture spacing of 5 mm (Fig. 2). In both groups, wound length will be measured prior to fascial closure, after which suturing will be performed. In the IS group, the number of sutures used will be counted. In the CBS group, the remaining suture length will be measured, and the suture length used for fascial closure will be calculated. In the CBS group, a SL:WL ratio (SL: WL) of 4:1 will be targeted.

### Implementation

In each participating institution, surgeons and operating room nurses involved in the trial will receive training through presentations and demonstration videos before the start of the study. Surgeons responsible for fascial closure via mini-laparotomy must be board-certified specialists. Prior to the initiation of the trial, they are required to perform both suturing techniques using a demonstration model, and only those who are certified as competent by the study coordinating center will be allowed to perform suturing within the trial. The trial at each center may proceed only once surgeons are trained in both techniques, nurses are trained in stitch and suture counting and measurement, and the study team has confirmed readiness. Throughout the study, unscheduled audits will be performed to monitor adherence to the protocol and to ensure quality control.

### Outcome parameters

#### Primary outcome

- The primary endpoint is the incidence of IH within 18 months after surgery, primarily assessed using scheduled abdominal CT imaging performed at predefined follow-up time points in all patients, with clinical examination serving as a complementary assessment.

#### Secondary outcomes

- Postoperative wound complications
- Reoperation or readmission
- Postoperative wound pain
- Fascial closure time

According to the definition proposed by the European Hernia Society (EHS), IH is defined as “any abdominal wall gap with or without a bulge in the area of a postoperative scar, perceptible or palpable by clinical examination or imaging.” [28] The EHS classification of IH will also be applied.

### Classification of incisional hernia

IH will be categorized according to its location, size, reducibility, and symptoms. The date of hospital discharge and the occurrence of postoperative complications will be recorded.

In accordance with the Japanese Guidelines for the Treatment of Colorectal Cancer [29], patients will undergo abdominal examinations, including palpation, every three months, and abdominal CT scans will be performed every six months. The following data will be collected at predefined time points.

#### Preoperative data

- Date of birth
- Height and weight
- Medical history (including diabetes, cardiac disease, and previous laparotomy)
- Preoperative radiotherapy or chemotherapy
- Preoperative or intraoperative administration of corticosteroids or immunosuppressive agents
- History of previous abdominal surgery
- Presence of abdominal wall hernias
- ECOG Performance Status

#### Operative data

- Clinical tumor findings (primary tumor location, clinical TNM (tumor-node-metastasis) classification (cTNM), clinical stage (cStage), bowel obstruction, perforation, or fistula)
- Surgical approach (laparoscopic or robot-assisted)
- Type of surgical procedure
- Extent of lymph node dissection (D2 or D3)
- Combined resection of other organs
- SL:WL ratio (continuous suture group)
- Number of stitches (IS group)
- Length of incision
- Fascial closure time
- Estimated blood loss
- Operative time
- Conversion to open surgery
- Use of prophylactic antibiotics
- Drain placement
- Administration of thromboprophylaxis
- Intraoperative complications (e.g., bowel injury, bleeding)
- Use of epidural analgesia
- Blood transfusion

#### Postoperative data

- Days to first flatus or bowel movement
- Date of initiation of oral intake
- Use of analgesics after postoperative day (POD) 5
- Date of discharge
- Reoperation
- Postoperative complications (classified according to the Clavien–Dindo system)
- Adverse events (graded according to CTCAE v5.0)

The presence or absence of adverse events will be assessed through medical interviews and physical examinations. In the event that an adverse event occurs, the following information will be recorded: the name of the event, date of onset and date of recovery, worst grade, outcome, seriousness (serious or non-serious), expectedness (expected or unexpected), details of treatment and dates of treatment, and whether rehospitalization was required.

### Outpatient follow-up

Follow-up visits will be conducted in accordance with the Japanese Guidelines for the Treatment of Colorectal Cancer [29].

#### Clinical evaluation

Outpatient visits will be scheduled at 1 month after surgery and every 3 months thereafter, including assessment for:

- Incisional hernia
- Surgical site infection
- Other wound-related complications
- Other abdominal wall hernias (non-incisional)

#### Laboratory and tumor marker tests

- Tumor markers (CEA and CA19-9)
- Laboratory blood tests, including complete blood count (white blood cell count, lymphocyte count, hemoglobin, and platelet count), albumin, and CRP

#### Imaging

Abdominal CT scans will be performed at 6, 12, and 18 months after surgery. Imaging parameters will be standardized across all participating institutions as follows:

- Slice thickness: 3 mm (5 mm permitted)
- Contrast enhancement: 100 s after injection
- Contrast dose: 600 mg/kg

### Data submission and storage

All CT images obtained at each institution will be anonymized by removing personal identifiers such as patient ID, institution name, and other identifying information. The anonymized data will then be stored on CD/DVD media and sent to the study coordinating center (Department of Gastroenterological Surgery, Nagoya City University). After evaluation, the data will be securely stored in the same manner as other study data.

### Evaluation of incisional hernia by abdominal CT

Two specialists (one radiologist and one gastrointestinal surgeon) at Nagoya City University will independently and blindly evaluate the CT images. In the event of disagreement between the two evaluators, a third specialist will adjudicate and make the final determination.

To avoid bias related to the suturing method, the evaluators will be blinded to treatment allocation, and allocation information will not be accessible to them.

IH will be assessed using the following parameters:

1. Presence or absence of IH (based on the EHS definition)
2. Size of the fascial defect
3. Type of hernia contents

The assessment criteria will follow the definition proposed by the European Hernia Society (EHS), which defines hernia as “any abdominal wall gap with or without a bulge in the area of a postoperative scar, perceptible or palpable by clinical examination or imaging” [30]. The study’s calendars are shown in Table 1.

**Table 1:** Study calendar of the HIBISCUS study

Time point	Enrollment	Allocation	Post-allocation					
			Surgery	POD 30	3 months	6 months	1 year	1.5 years
Informed consent	●							
Eligibility screen	●							
Patient background	●							
Blood test	●			●	●	●	●	●
CT	●					●	●	●
Colonoscopy							●※	
Allocation		●						
Surgery			●					
Surgical outcome			●					
Intraoperative adverse event			●					
Physical examination	●			●	●	●	●	●
Pathological findings				●				
Early complications				●				
Readmission				●	●	●	●	●
Adjuvant therapy					●	●	●	
Late complications					●	●	●	●
Survival						●	●	●
Resurrence						●	●	●

※Colonoscopy is generally performed 1 year postoperatively, but may be advanced to 6 months if complete evaluation was not possible due to preoperative bowel obstruction

### Sample size calculation

Regarding the primary endpoint of IH, previous reports in laparoscopic surgery have shown an incidence of approximately 5–13% in patients undergoing conventional IS closure [17, 19–21]. Based on these data, an incidence of 11.5% was selected as a representative estimate for the IS group in the present trial.

In contrast, Pyo et al. reported an IH rate of 1.3% at 12 months following fascial closure with a monofilament polydioxanone barbed suture (MONOFIX^®^) in a prospective single-arm trial [22]. In our institution, the 1-year postoperative incidence of IH was 3.5% among 215 consecutive cases performed between June 2022 and December 2023.

Based on these previous findings, the incidence of IH in the CBS group was hypothesized to be reduced by approximately two-thirds compared with IS, resulting in an estimated incidence of 3.9%. With a two-sided significance level of 5% and a statistical power of 80%, the required sample size calculated using a two-sided chi-square test for proportions was 192 patients per group (total 384).

Allowing for an anticipated 10% dropout rate, the final target sample size was set at 422 patients. The calculation was performed using Stata software, version 17.0 (StataCorp, College Station, TX, USA).

### Statistical analysis

Continuous variables will be summarized as mean ± standard deviation (SD) if approximately normally distributed, and as median with interquartile range otherwise; categorical variables will be presented as counts and percentages. Baseline characteristics will be evaluated descriptively without formal hypothesis testing, in accordance with standard recommendations for randomized controlled trials, which discourage statistical testing of baseline variables. All analyses will be performed according to the intention-to-treat (ITT) principle.

For comparisons between groups, t-tests or Mann–Whitney U tests will be applied to continuous variables depending on the distribution assessed using the Kolmogorov–Smirnov test with Lilliefors correction. Categorical variables will be compared using chi-square tests or Fisher’s exact tests, as appropriate.

The primary endpoint of this RCT is the incidence proportion of IH at the mini-laparotomy site. Differences between groups will be evaluated using a two-sided chi-square test for proportions with a two-sided significance level of 5%, based on the prespecified hypothesis that CBS reduces the risk of IH compared with IS. The primary analysis population will include all randomized participants according to the ITT principle. A per-protocol set (PPS) analysis will be conducted as a sensitivity analysis including those who complete the allocated intervention according to the protocol.

Secondary endpoints—including intraoperative outcomes, early postoperative outcomes, the incidence of intraoperative complications, and readmission within 90 days after surgery—will be summarized descriptively for each group.

For the primary endpoint analysis, the incidence proportion of IH during the follow-up period and the corresponding 95% confidence intervals will be calculated for each group. In addition, the odds ratio and 95% confidence interval for IH incidence in the CBS group relative to the IS group will be estimated.

### Monitoring

Monitoring will be conducted to confirm the progress of the study and to ensure that it is implemented in accordance with the Ethical Guidelines and the approved study protocol. The principal investigator will appoint monitoring staff, who will be responsible for preparing and submitting monitoring reports to the study coordinating center. Monitoring staff will review source documents, including medical records and case report forms, approximately every six months from the date of the first patient registration. They will assess the following monitoring items and prepare a monitoring report.

The study representative will also prepare a central monitoring report approximately every six months from the start of enrollment, based on data aggregated from each participating institution.

### Central monitoring items

- Registration status (number of enrolled cases across all institutions)
- Case information (baseline characteristics, study progress, details of discontinued cases)
- Case report form (CRF) entry status, including query management
- Deviations from the study protocol
- Other issues affecting study progress

Following each site monitoring visit, the monitoring staff will promptly prepare and submit a site monitoring report.

#### Site monitoring items

- Registration status (number of cases enrolled at the site)
- Eligibility assessment for each registered case
- Presence or absence of serious adverse events
- Presence or absence of major protocol deviations
- Other site-specific issues related to study progress, safety, or data reliability
- Compliance with relevant ethical guidelines

## Discussion

This RCT aims to evaluate whether CBS is superior to IS in reducing the incidence of IH in patients undergoing laparoscopic or robot-assisted colorectal surgery. In recent years, advances in suture technology and the widespread adoption of minimally invasive and robotic colorectal surgery have renewed interest in optimizing fascial closure techniques for small midline incisions. However, despite emerging experimental and clinical data suggesting the safety and potential advantages of barbed sutures, high-quality randomized evidence in this specific surgical context remains limited.

Standardizing surgical technique is a major challenge in studies evaluating fascial closure methods, particularly in multicenter trials where surgeons with different levels of experience may contribute to variability. To minimize such variability, only surgeons who successfully completed a pre-trial competency assessment using a synthetic abdominal wall bench model were permitted to perform fascial closure, ensuring technical uniformity across all participating centers. Nevertheless, learning-curve effects and inter-center variability may still have influenced the results, and these factors should be considered when interpreting the findings.

The rationale for selecting an 8-mm bite width and 8-mm suture spacing for IS and a 5-mm bite width and 5-mm spacing for CBS is based on both existing evidence and prevailing clinical practice. For CBS, the 5-mm/5-mm parameters were chosen according to the STITCH trial, which demonstrated that a 5-mm bite width with 5-mm spacing significantly reduced the incidence of IH compared with a 10-mm bite width and 10-mm spacing protocol [30]. For IS, a nationwide survey of 50 institutions showed that 8-mm bites and 8-mm spacing were the most commonly used settings in Japan. After detailed discussion among all participating centers, these parameters were adopted to reflect real-world surgical practice and to enhance the pragmatic nature and generalizability of the trial.

In addition, the use of different suture calibers between the two groups warrants clarification. In this trial, a size 1 barbed suture was used for continuous closure, whereas a size 0 monofilament suture was used for interrupted closure. This decision was not intended to introduce differences in tensile strength or biomechanical performance between the two techniques. Rather, it reflects product-specific characteristics and prevailing clinical practice patterns. The same nationwide survey demonstrated that size 1 sutures are most commonly used for continuous fascial closure, whereas size 0 sutures are predominantly used for interrupted closure in Japan. After careful discussion among all participating centers, these suture calibers were selected to align with routine clinical usage and to preserve the external validity of the trial. Nonetheless, we acknowledge that the use of different suture sizes represents a potential limitation and should be taken into account when interpreting the results.

Standardizing bite width and suture spacing is essential in RCTs comparing IS and CBS because such standardization strengthens reproducibility and internal validity by minimizing inter-institutional and inter-surgeon variations. Moreover, this trial may serve as a standardized model for future international studies and contribute to global optimization of fascial closure strategies.

If CBS is shown to be superior to IS in reducing IH, CBS could become the preferred fascial closure method in minimally invasive colorectal surgery. Conversely, if CBS does not demonstrate superiority, IS would remain the standard approach, and the routine use of CBS may not be recommended. Regardless of the outcome, current evidence comparing CBS and IS remains limited and heterogeneous; therefore, this trial is expected to make an important contribution to evidence generation in this field and to inform future clinical guidelines and surgical practice.

## Conclusion

The HIBISCUS trial is a multicenter randomized controlled study designed to compare CBS with IS for midline fascial closure in mini-laparotomy incisions during laparoscopic and robot-assisted colorectal surgery. By rigorously standardizing suturing parameters and ensuring that only board-certified surgeons perform fascial closure, this trial aims to generate high-quality evidence regarding an optimal technique to reduce the incidence of IH and other wound-related complications. Given the current lack of robust comparative data between these two suturing methods, the results of this trial—regardless of outcome—are expected to provide valuable evidence that will help establish evidence-based best practices for abdominal wall closure. Furthermore, the HIBISCUS trial is expected to yield important findings generated in Japan that can be disseminated internationally and contribute to the global optimization and standardization of fascial closure techniques.

## Supplementary Information

Supplementary Material 1.

## Data Availability

Trial registration: This study is registered in the Japan Registry of Clinical Trials (jRCT1040250129). Registration date: November 21, 2025. The datasets used and/or analysed during the current study are available from the corresponding author on reasonable request.

## Abbreviations

IH: Incisional hernia
RCT: Randomized controlled trial
ECOG: Eastern Cooperative Oncology Group
EDC: Electronic data capture
SL:WL: Suture length to wound length ratio
SSI: Surgical site infection
EHS: European Hernia Society
ITT: Intention-to-treat
PPS: Per-protocol set
