# Supplementary material for: HIBISCUS trial (hernia incidence with continuous barbed vs interrupted suturing in colorectal surgery with small incisions): a contemporary study protocol for a multicenter randomized controlled trial
Source: BMC Surg. 2026 Jan 26;26:151. doi: 10.1186/s12893-026-03528-5 (PMC12918296; doi:10.1186/s12893-026-03528-5)
Supplement: Supplementary file 1 — Supplementary Material 1. [file 12893_2026_3528_MOESM1_ESM.docx]

# SPIRIT 2013 Checklist (2025-style) – HIBISCUS Trial

| Section | Item | Description | Manuscript  Page |
| --- | --- | --- | --- |
| Administrative | 1 | Title | p.1 |
| Administrative | 2a | Trial registration | p.6,14,38 |
| Administrative | 2b | Protocol version | p.3 |
| Administrative | 3 | Funding | p.39 |
| Administrative | 5a | Contributors | p.1–3,38 |
| Administrative | 5b | Sponsor contact | p.3,39 |
| Administrative | 5c | Role of sponsor | p.39 |
| Administrative | 5d | Committees/DMC | p.32–33 |
| Introduction | 6a | Background & rationale | p.7–12 |
| Introduction | 6b | Comparator rationale | p.10–12,34–35 |
| Introduction | 7 | Objectives | p.12–13 |
| Methods | 8 | Trial design | p.13–14 |
| Methods | 9 | Study setting | p.14 |
| Methods | 10 | Eligibility criteria | p.16–17 |
| Methods | 11a | Interventions | p.19–21 |
| Methods | 11b | Discontinuation/modification | p.18,32–33 |
| Methods | 11c | Adherence | p.20–21,32–33 |
| Methods | 12 | Outcomes | p.13,21–27 |
| Methods | 13 | Timeline | p.28 |
| Methods | 14 | Sample size | p.29–30 |
| Methods | 15 | Recruitment | p.14,16 |
| Assignment | 16a | Sequence generation | p.18–19 |
| Assignment | 16b | Allocation concealment | p.19 |
| Assignment | 16c | Implementation | p.18–20 |
| Blinding | 17a | Who blinded | p.27 |
| Blinding | 17b | Unblinding | p.27 |
| Data | 18a | Data collection | p.22–26 |
| Data | 18b | Retention | p.25–28 |
| Data | 19 | Data management | p.18,26–27 |
| Data | 20a | Statistical methods | p.30–31 |
| Data | 20b | Additional analyses | p.31 |
| Data | 20c | Analysis population | p.30–31 |
| Data | 20d | Missing data | p.30–31 |
| Monitoring | 21a | Data monitoring | p.32–33 |
| Monitoring | 21b | Interim analysis | p.32 |
| Monitoring | 22 | Harms | p.24–25 |
| Monitoring | 23 | Auditing | p.32–33 |
| Ethics | 24 | Ethics approval | p.14,39 |
| Ethics | 25 | Protocol amendments | p.14 |
| Ethics | 26a | Informed consent | p.16–17,39 |
| Ethics | 26b | Additional consent | p.16–17 |
| Ethics | 27 | Confidentiality | p.26–27 |
| Ethics | 28 | Competing interests | p.39 |
| Ethics | 29 | Access to data | p.38 |
| Ethics | 30 | Post-trial care | p.39 |
| Ethics | 31a | Dissemination | p.37–39 |
| Ethics | 31b | Authorship | p.38–39 |
| Appendix | 32 | Consent materials | p.16–17 |
| Appendix | 33 | Biological specimens | N/A |
